# Systematic review of racial and ethnic disparities pertaining treatment in mental healthcare amongst incarcerated patients

**DOI:** 10.1192/j.eurpsy.2021.1887

**Published:** 2021-08-13

**Authors:** A. Quintão, M.D. Urzal, I. Donas-Boto, M.M. Lemos, F. Coelho, H. Simião, N. Moura, J. Vian

**Affiliations:** 1 Psychiatry Department, Ocidental Lisbon Hospital Center, Lisboa, Portugal; 2 Psychiatry, Centro Hospitalar de Lisboa Norte, Lisbon, Portugal

**Keywords:** ethnic, disparities, racial, incarcerated

## Abstract

**Introduction:**

Research has shown that ethnic/racial minorities have a higher risk of homelessness, involvement with the criminal system, psychiatric misdiagnosis, treatment delay, and being prescribed first (versus second) generation antipsychotics.

**Objectives:**

To investigate if the disparities found in the community are replicated in incarcerated patients.

**Methods:**

Systematic review on PubMed for articles that fulfilled criteria for 4 domains: prison, psychosis, race/ethnic, and treatment.

**Results:**

Forty-one articles matched the search criteria. Of those, 24 were irrelevant; 2 were inaccessible. Fifteen articles were considered; most highlighted the interplay between the criminal system, homelessness, mental disorders, and ethnic/racial minorities. Five articles highlighted differences in treatment. One stated that African-Americans and Asians were less likely than Whites to have access to mental health services. Concerning treatment for substance use disorders, one study found Hispanic inmates were more often engaged in treatment, followed by Caucasians and lastly, African-Americans; a different study reported the percentage of Whites and Blacks receiving treatment was similar, while Latinos were under-represented. Whites were most likely to have mental health counseling/substance use treatment as part of their sentence. A study from New-Zealand stated that treatment for mental disorders was less common for Maoris, in whom suicidal thoughts were often unrecognized. The last study reported a higher risk of self-harm for foreign patients, coupled with non-recognition/misinterpretation of symptoms.

**Conclusions:**

Racial/ethnic inequalities show that disparities in healthcare are pervasive in all settings. More studies are needed to better understand the complex nature of this problem.

**Disclosure:**

No significant relationships.

